# Long-Term Natural Course of Pathologic Myopia in Chinese Patients

**DOI:** 10.1155/2019/1210398

**Published:** 2019-03-19

**Authors:** Minjie Chen, Manrong Yu, Jinhui Dai, Renyuan Chu

**Affiliations:** ^1^Department of Ophthalmology, Eye and ENT Hospital of Fudan University, Shanghai, China; ^2^NHC Key Laboratory of Myopia (Fudan University), Laboratory of Myopia, Chinese Academy of Medical Sciences, Shanghai, China

## Abstract

**Purpose:**

To investigate the natural progression in Chinese patients with pathological myopia (PM) and its associated factors.

**Methods:**

The medical records of 28 patients with PM (worse than −6 diopter), including 31 eyes of 21 children and 12 eyes of 7 adults, were studied. All of the patients underwent a complete ophthalmologic examination at least twice over 3-year period, including the measurement of refractive error (shown as spherical equivalent, SE), axial length (AL), intraocular pressure, visual acuity (uncorrected visual acuity, UCVA, and best-corrected visual acuity, BCVA), and dilated fundus examination.

**Results:**

The median AL of adults increased significantly from 29.8 mm to 31.43 mm over 5.4 years follow-up (*P*=0.0037), accompanied with the median SE progressing significantly from −16.4 D to −18.94 D (*P*=0.0005). Similarly, the median AL of children increased significantly from 26.13 mm to 27.81 mm over 3.9 years (*P*=0.0001). However, the improvements of UCVA and BCVA in children were significant (*P*=0.0304, 0.0001), and they had a negative correlation with age (*P*=0.0010, 0.0005). Also, UCVA and BCVA in children with bilateral PM were significantly better than those with unilateral PM (*P*=0.0385, 0.0210).

**Conclusions:**

Fundus degenerations in children with pathological myopia may lead its way since the age of 10 years. Besides, children with bilateral pathological myopia can have parallel development in visual acuity.

## 1. Introduction

Myopia has emerged as a major public health concern nowadays with the striking evidence existing for rapid increases in its prevalence [1]. Among which, interethnic comparisons showed that the incidence of myopia was higher in Chinese than in non-Chinese [2–5]. The similar findings observed in the Correction of Myopia Evaluation Trial (COMET), a randomized double-masked multicenter clinical study, identified that Asian populations in America experienced a greater prevalence of myopia [6]. A multicenter observational study, which included 4 ethnic groups from 4 different locations in the United States, also found Asian children tend to have the highest prevalence of myopia, compared with Hispanics and African Americans [7]. Therefore, ethnicity may play an important role in myopic prevalence even in its progression.

With the increasing prevalence of myopia in most populations worldwide, the prevalence of pathological myopia (PM) has also increased. Pathological myopia, clinically characterized by continuous eye elongation, resulting from scleral thinning and posterior staphyloma, initiates serious intraocular complications, and leads to varying degrees of visual deterioration [8, 9]. The prevalence of PM is also known to be different among races, more common in the adult Asian population, at approximately 9% [10], than in the Caucasians in the United States at 2% [5]. In addition, PM was the second most common cause of low vision and blindness for people aged 40 years and older Chinese individuals [11] and was also reported to be the leading cause of visual impairment [12–14].

However, little is known about the natural history of pathological myopia. A PubMed search extracted no more than 5 articles describing a natural course of PM, and none of which was conducted in Chinese population [15–18]. Considering the interethnic difference, the purpose of this study was to investigate the natural changes of axial length, refraction, and intraocular complications in Chinese patients with PM, as well as the potential associated factors.

## 2. Patients and Methods

The study followed the tenets of the Declaration of Helsinki (1964) and was approved by Ethics Committee of Eye and ENT Hospital of Fudan University. Since this was a retrospective study with no interventions involved, patients or their guardians were acknowledged with the study protocol verbally without signing written consent form.

The medical records of consecutive patients with PM who were examined in Eye and ENT Hospital of Fudan University from 2008 through 2012 and a minimum follow-up of 2 years were retrospectively analyzed. Eligible subjects were diagnosed with PM accompanied with the following criteria: refractive error should be worse than −6.0 D (spherical equivalent, SE) when patients were younger than 8 years old, worse than −8.0 D when aged between 8 and 12 years old, worse than −10.0 D when aged between 12 and 18 years old, and worse than −12.0 D when older than 18 years old; and the best-corrected visual acuity (BCVA) should be no better than 20/20. The exclusion criteria were (1) nonspectacles myopia corrections, such as contact lenses, pharmaceuticals, and surgeries; (2) history of vitreoretinal surgery or cataract surgery; (3) moderate to severe cataract obstructing an accurate measurement of the axial length; (4) any history of nystagmus, glaucoma, lens abnormality, or retinal disorders which would influence axial length measurement or fixation; and (5) active choroidal neovascularization at the initial examination or during the follow-up, where exudative changes such as serous retinal detachment would change axial length.

All of the patients underwent a complete ophthalmologic examination at each visit, including the measurement of the refractive error (spherical equivalent), axial length (AL), intraocular pressure (IOP), visual acuity, and dilated fundus examination. AL was measured using noncontact IOLMaster (Carl Zeiss Meditec AG, Jena, Germany) at least 5 times for each eye at each examination and was averaged for statistical analyses. The uncorrected visual acuity (UCVA) was examined with a standard logarithmic visual acuity chart. Subjective refraction of children was examined by experienced optometrists using autorefractor (ARK-700A autorefractor and SSC-330 scientific subjective refractor, NIDEK, Japan) half an hour after cycloplegia induced with three drops of 0.5% tropicamide with five-minute intervals. Refractive error examination for children younger than 3 years old was conducted using retinoscopy after cycloplegia. IOP was tested at least 3 times with Canon TXF-noncontact tonometer and averaged for analysis. All technicians were blind to the study.

### 2.1. Statistical Analysis

All statistical analyses were performed with Stata software version 11.0 (Stata Corp., College Stations, TX, USA). Visual acuity was converted to logMAR for data analysis. Changes within groups were analyzed using the Wilcoxon signed-rank test. Comparisons between the groups were performed with Student's *t*-test or the Mann–Whitney rank-sum test. In addition, factors that might be associated with the AL increase and myopic progression were analyzed using regression analysis. *P* value < 0.05 was considered statistically significant.

## 3. Results

Of all the eligible medical records, 43 eyes of 28 patients met the inclusion criteria, where 14 patients were diagnosed with unilateral PM and 21 patients (31 eyes) were younger than 18 years. The clinical characteristics of children and adults at the initial examination are shown in Table 1.

During the follow-up period, the median AL increased significantly from 26.13 mm at the initial visit to 27.81 mm at the final examination in the 31 eyes of children (*P*=0.0001), equivalent of 1.68 mm axial elongation over 3.9 years (0.43 mm per year). Among which, 18 eyes (58%) experienced an axial elongation no more than 2.0 mm during the period, equal to 0.5 mm per year, while 7 eyes (23%) elongated more than 2.0 mm. Compared to children, the median AL in the 12 eyes of adults increased significantly from 29.8 mm to 31.43 mm during the follow-up (*P*=0.0037), equal to 1.63 mm axial elongation over a period of 5.4 years (0.30 mm per year). Six eyes (50%) experienced an axial elongation no more than 1 mm during the course, equal to 0.2 mm per year; among which, 5 eyes (42%) elongated no more than 0.5 mm (0.1 mm/year). However, 4 eyes (33%) of adults had an axial elongation more than 2.0 mm.

In terms of refractive error, 31 eyes of children experienced a median myopia progression of −3.35 D during the 3.9-year follow-up, ending up with −12.25 D at the final visit (*P*=0.0001), equal to 0.86 D progression per year. Of all the children, 3 eyes (10%) remained stable (no more than 0.5 D), whereas 8 eyes (26%) progressed significantly by more than 4 D. As for the refractive changes in adults, the median SE changed significantly from −16.4 D at the first visit to −18.94 D at the last examination in all 12 eyes (*P*=0.0005), equal to −2.54 D over 5.4 years of follow-up (−0.47 D per year). Among which, only one eye was stable (no more than 0.5 D), while two eyes (17%) progressed more than 4 D.

**Figure 1:**
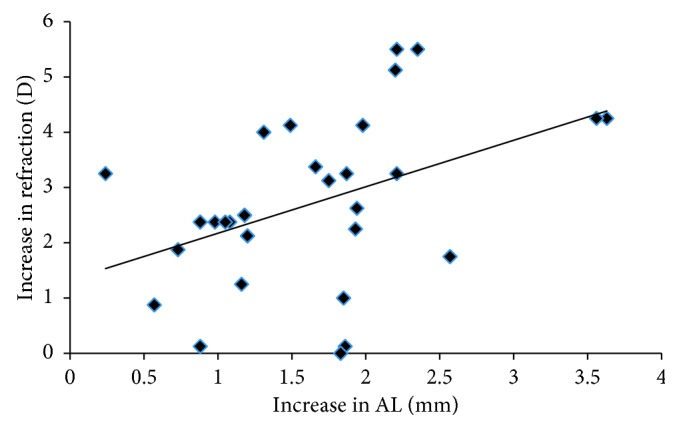
The correlation between myopia progression and axial elongation during the follow-up period in children. AL: axial length; D: diopter.

**Figure 2:**
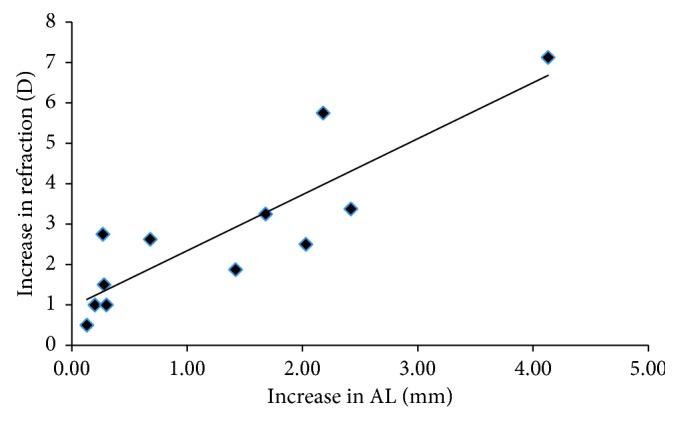
The relationship between myopia progression and change in axial length during the follow-up period in adults. AL: axial length; D: diopter.

As we expected, myopia progression had a significantly positive relationship with the increase of AL for both children and adults (*P*=0.0136, 0.0002) (Figures 1 and 2). However, neither AL nor the IOP at the initial visit was significantly correlated with the increase of AL (*P* > 0.05). For children, axial elongation and myopic progression had no association with the age at their initial visits (*P*=0.0840, 0.8711). However, the myopic progression was negatively correlated with the SE at the initial visit (*P*=0.0013).

With regard to the IOP, no significant difference was found in both children and adults at the final visit when compared to that at the first visit (*P*=0.1325, 0.8377). But for visual acuity, significant improvement was observed in both UCVA and BCVA of children at the final visit, compared to those of the initial visit (*P*=0.0304, 0.0001). However, deterioration of UCVA was found in adults during the follow-up (*P*=0.0293), whereas BCVA remained unchanged (*P*=0.1545). Children were divided into four groups according to the age at the initial visit (Table 2). In general, the improvement of both UCVA and BCVA was negatively correlated with age (*P*=0.0010, 0.0005). Three younger groups of children experienced significant improvement of UCVA but children older than 10 years old had UCVA deterioration (Table 2). In addition, BCVA improvement was observed in two younger groups but not in two older groups (Table 2), suggesting that children with extensive myopia older than 10 may have little opportunity in BCVA improvement.

**Figure 3:**
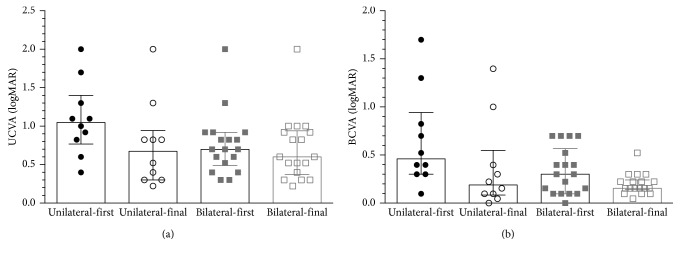
Comparison of visual acuity ((a) UCVA and (b) BCVA) between children with unilateral pathological myopia and bilateral myopia at their first and final visits. UCVA: uncorrected visual acuity; BCVA: best-corrected visual acuity; logMAR: Logarithm of the Minimum Angle of Resolution.

Children were also grouped by unilateral or bilateral PM, and the clinical characteristics of the initial examination are shown in Table 3. UCVA and BCVA were significantly better in the bilateral group (*P*=0.0385, 0.0210) despite that the refractive error and AL were comparable between the two groups. During the follow-up period, no significant between-group difference was found in the axial elongation or myopic progression (*P*=0.7981, 0.5631). However, UCVA of the unilateral group improved more than the bilateral group (*P*=0.0344, Table 3, Figure 3), as well as the BCVA, though not significantly (*P*=0.0557).

## 4. Discussion

Although more and more pathological myopia patients have been visiting our clinics, we had rare chance to follow the natural course of PM since high myopia and related complications were usually treated, preventing us from enrolling more cases in our study. However, to our knowledge, it was the first report focusing on the natural progression of PM in Chinese patients as well as the first study concerning the natural course of PM development in children.

It was acknowledged that AL would reach adult length and remained stable by the age of 13 [19, 20], it was not the case for patients with progressive PM. Saka et al. reported that the median increase of AL in high myopic eyes with and without various pathologic changes was 0.08 mm per year (0.06 mm/year in the age group of <45 years and 0.12 mm/year in the age group of ≥45 years [15]), as determined by A-scan ultrasound. In another study, Takahashi et al. used IOLMaster to determine the mean AL increase by 0.085 mm per year [21]. In the current study, the median axial elongation per year was about 0.3 mm in adults, much longer than that reported by previous studies. Even with the acknowledged disparity of axial measurement 0.09 mm longer with IOLMaster than ultrasound [22], a 0.21 mm/year-axial elongation difference still remained in our study, which may be resulted from the different inclusion criteria. In the previous studies, the definition of high myopia was refractive error equal to or worse than −6.0 diopters (D) or AL ≥ 26.5 mm. However, adult patients enrolled in the current study had a refractive error of −12.0 D or more, much more myopic than previous studies. It was acknowledged that PM was characterized by progressive axial elongation and myopia development. Thus, our results may reflect that the axial elongation increased with the degree of PM, or Chinese PM patients may be more susceptible to axial elongation. Since the studies of Saka and Takahashi were both conducted in Japan, another high-prevalence area, interethnic progression of AL in PM also existed. In the western area, Fledelius and Goldschmidt reported a significant mean AL increase, from 26.7 ± 1.3 mm at age 26 to 27.5 ± 2.1 mm at age 54, in 39 high myopic patients, equivalent to 0.03 mm elongation per year [22]. The much longer follow-up periods may be responsible for the obvious difference. Nevertheless, these findings suggest that further studies warrant to determine whether the myopia progression was affected by ethnic difference.

Numerous studies focused on myopia progression in children, but neither of them paid attention to the progression of PM to date. In the current study, the median AL growth was 1.68 mm over 3.9-year follow-up (0.43 mm/year) in children, equal to a 0.86 D myopic progression per year. However, Saw et al. reported a myopia progression rate of −2.40 D in 7-year old, −1.97 D in 8-year old, and −1.71 D in 9-year old during 3 years, about −0.8 D per year in 7-year old children [23]. Also, in a Hong Kong myopia study of school children aged 5 to 16 years, the myopia progression rate was −0.63 D per year [24], slightly less than the rate in the current study. As for the AL elongations, Saw et al. reported an average increase of 0.89 mm over 3 years [25], similar to the myopic children aged 7–10.5 years (*n*=133) in the Hong Kong study who showed AL increase by 0.32 mm per year (0.96 mm over 3 years) [26]. In contrast, in the COMET trial, the rate of axial elongation was slightly lower in myopic children aged 6–11 years which was 0.75 mm over 3 years [27]. Previous studies have demonstrated an evident greater rate of myopia progression in Asian children than in age-matched European children [23, 27], but none of them defined the PM. Now, we deemed that the Chinese pathological myopic children experienced faster axial elongation but similar myopic development annually compared to children with mild to moderate myopia. Besides, the negative correlation between the myopic progression and the refractive error at the initial visit was different from previous studies. PM in children was mostly infantile-onset myopia and considered to be associated with the onset of amblyopia [28, 29]. Since the lazy eye may not be so engaged in visual activity, the myopic development could be slowed down to some extent. Considering the difference between bilateral and unilateral PM, the former performed much better in visual acuity than the latter one. Visual acuity was usually worse in the pathological myopic eye than the fellow eye in unilateral myopia and had unbalanced development interocularly, but improved simultaneously between the two eyes in bilateral PM. However, no notable difference was observed in axial elongation or myopia progression between the two groups.

It is well known that the change of refractive error is associated with the elongation of eyeball. Our data were consistent with previous results that the myopia progression was positively correlated with the increase of AL both for children and adults. The result that AL elongation had no relationship with axial length at the initial examination or IOP was in agreement with Saka's research [15]. Also, no correlations between the age and AL elongation or myopia progression in children were demonstrated.

In comparison with the deteriorated UCVA in adult pathological myopes, UCVA and BCVA in PM children improved during the follow-up period. Posterior staphyloma and fundus degenerations are not common in highly myopic children [30], but the incidence and severity of pathological changes in high myopia increase with age [31]. The improvement of visual acuity in highly myopic children may be resulted from ongoing visual development as well as mild pathological features. In addition, children younger than 10 years showed improvement of UCVA, while their older counterpart showed deteriorations. These findings suggest that the visual acuity of infantile-onset PM eyes may first improve with ocular development; then at a certain age, it deteriorates due to that the incidence and severity of pathological disorders increase with age, and the certain age may be 10 years of age according to our results. Though the dividing age was not clear in BCVA, the consistent trend of visual acuity development suggested that the certain age would probably be older than 10 years old, while this hypothesis needs further confirmation. The insufficient sample was a limitation, so a larger population is necessary to extract the exact age when fundus deterioration appears in highly myopic children. Moreover, this retrospective study was limited by the existing medical records, providing at most 7-year follow-up.

## 5. Conclusions

In conclusion, this is the first study reporting the natural progression in Chinese patients with PM and its possible associated factors, in which we demonstrated a 1.68 mm median axial elongation over 3.9 years, accompanied with a −3.35 D myopia progression in children, whereas in adults, it was 1.63 mm over 5.4 years and −2.54 D. However, neither the AL at the initial examination nor the IOP was significantly correlated with the increase in the AL. Children with PM had their UCVA and BCVA improved with age before 10 years old, whereas fundus degeneration may lead visual acuity deteriorations thereafter. Besides, children with bilateral PM showed better performance in visual acuity than their unilateral counterparts.

## Figures and Tables

**Table 1:** Clinical characteristics of children and adults at the initial examination.

	Children (31 eyes of 21 patients)	Adults (12 eyes of 7 patients)
Median age at initial examination (range) (yrs)	6.6 (1.1 to 14.3)	38.1 (25.7 to 45.6)
Median SE (range) (D)	−8.9 (−6 to −21.5)	−16.4 (−12.5 to −22.5)
Median axial length (range) (mm)	26.13 (21.53 to 30.11)	29.8 (27.16 to 32.35)
Median follow-up (range) (yrs)	3.9 (2 to 7.2)	5.4 (2.4 to 6)

D: diopters; yrs: years; SE: spherical equivalent.

**Table 2:** Comparison of visual acuity (UCVA and BCVA separately) of children at different ages between the first and last visits.

Grouping by age (yrs)	<4 (*n*=6)	≥4 and <7 (*n*=9)	≥7 and <10 (*n*=9)	≥10 (*n*=7)
Follow-up periods (yrs)	3.23 ± 0.90	3.91 ± 1.06	5.20 ± 1.53	3.74 ± 1.84
*P* value for UCVA	0.0063	0.0465	0.0404	0.0202
*P* value for BCVA	0.0284	0.0076	0.5733	0.5292

*n*: number of eyes; yrs: years; UCVA: the uncorrected visual acuity; BCVA: best-corrected visual acuity.

**Table 3:** Comparison of clinical characteristics between bilateral and unilateral pathological myopia in children at the initial visit.

	Unilateral PM (11 eyes of 11 children)	Bilateral PM (20 eyes of 10 children)	*P* value
Median age at initial examination (yrs)	5.5	8.9	0.1286
Median SE (D)	−7.25	−9.19	0.0630
Median axial length (mm)	25.94	26.53	0.1795
Median UCVA at initial visit (logMAR)	1.05	0.70	0.0385
Median UCVA at final visit (logMAR)	0.67	0.60	0.772
Follow-up (range) (yrs)	3.9	3.8	0.5356

yrs: years; SE: spherical equivalent; PM: pathological myopia; UCVA: uncorrected visual acuity.

## Data Availability

The data used to support the findings of this study are available from the corresponding author upon request.
